# Anatomy

**Published:** 1875-06

**Authors:** E. S. Holmes


### ANATOMY.

BY E. S. HOLMES.

*Read before the Michigan Dental Society.*

To make a report on a subject as dry and prosy as anatomy without being pedantic, would seem to be difficult, even before a nonprofessional audience, and impossible when the listeners are supposed to be as well or better acquainted with the subject than the speaker. I shall not therefore attempt to make a regular report, but try to give something that will induce others to follow with remarks and facts that will be more to the point and better than anything I can present.

Anatomy may be defined in the general way as "the science of the form and structure of organic bodies, and a knowledge of it is practically acquired by the separation of the parts of a body, so as to show their distinct formation, and their relations with each other." An accurate knowledge of which is indispensable to the curist, the surgeon and to all who are called upon to treat diseases either wholly or in part by manual op, erations.

According to Prof. Dunglison's Medical Dictionary a dentist is "is one who devotes himself to the study of the diseases of the teeth, and their treatment." Or to give a fuller and more comprehensive definition, I should say a dentist is one who is acquainted with the anatomy, physiology and pathology of the organs of mastication, and devotes himself to the treatment and cure of their diseases, and of the diseases of those parts of the human organism depending upon and produced by their abnormal condition.

Allowing these definitions to be correct we see the absolute necessity of the dentist being a good anatomist. The teeth are very important accessories to the digestive apparatus, the front gate of the citadel of nutrition. The dentist to be worthy of the name must be intimately acquainted not only with the anatomy of the teeth, but with that of the whole system of which they form so important a part. Anatomy is the foundation stone on which the art of cure is constructed, aided by physiology, pathology and diagnosis. How often it occurs, that a disease is incorrectly diagnosed and treated for months without avail for the want of definite anatomical knowledge. A diseased condition of the whole alimentary canal may be caused in consequence of a primary ailment of the organs of mastication. In such cases, all treatment must be only palliative, which does not remove the producing cause. Again, an intimate knowledge of the nervous system, and particularly the nerves that are immediately connected with the teeth and mouth is absolutely essential to enable the dentist to serve his patients faithfully and well. Pseudo-neuralgia that has baffled the skill of one not so well posted, for months or years, has often been cured by the well educated and judicious dentist in a few minutes, and perhaps by a single application of medicine.

It is also equally important to understand the muscles, their attachments and their line of action. Abscesses with external fistulous openings so far removed from the mouth that their origin or cause would never have been discovered by superficial anatomical knowledge, have been traced by the course of muscles to necrosed roots of teeth and then, of course, easily cured. And so we might speak of the other tissues. But enough has been said to call to our minds the superlative importance of a thorough anatomical knowledge. This is all I hope to do on this occasion. And I hope that all who are tutors will see to it that their students are well posted in anatomy, insist upon it, and instill into their minds the fact that without a thorough knowledge of anatomy, their professional acquirements must necessarily be superficial.

